# Incidence and Impact of Ventilator Associated Multidrug Resistant Pneumonia in Patients with SARS-COV2

**DOI:** 10.1155/2022/9730895

**Published:** 2022-09-02

**Authors:** Seife Yohannes, Zaki Ahmed, Rachel Schelling, Swaminathan Perinkulam Sathyanarayanan, Alexandra Pratt, Mathew P. Schreiber

**Affiliations:** ^1^Department of Critical Care Medicine, MedStar Washington Hospital Center, Washington, DC, USA; ^2^Georgetown University School of Medicine, Washington, DC, USA; ^3^Department of Internal Medicine, University of South Dakota Sanford School of Medicine, Vermillion, SD, USA

## Abstract

**Introduction:**

Ventilator Associated Pneumonia (VAP) is associated with significant cost, morbidity, and mortality. There is limited data on the incidence of VAP, appropriate antibiotic timing, and the impact of multidrug resistant VAP in intubated Coronavirus disease-19 (COVID-19) patients.

**Methods:**

A retrospective study was conducted at 2 tertiary urban academic centers involving 132 COVID-19 patients requiring invasive mechanical ventilation (IMV). The epidemiology of VAP, the impact of prior empiric antibiotic administration on the development of Multidrug Resistant Organism (MDRO) infections, and the impact of VAP on patient outcomes were studied.

**Results:**

The average age of the patients was 60.58% were males, 70% were African-Americans and two-thirds of patients had diabetes, hypertension, or heart disease. The average Body Mass Index (BMI) was 32.9. Forty-one patients (27%) developed VAP. Patients with VAP had a significantly higher Sequential Organ Failure Assessment (SOFA) score prior to Intensive Care Unit (ICU) admission. Sixty percent received empiric antibiotics before initiation of IMV, mostly on hospital admission, and 81% received empiric antibiotics at the time of intubation. The administration of empiric antibiotics was not associated with a higher prevalence of VAP. The prevalence of VAP was 22 per 1000 days on ventilation. No difference in mortality was seen between VAP and non-VAP groups at 49% and 57% respectively (*p* = 0.4). VAP was associated with increased ICU length of stay (LOS), 30 vs. 16 days (*p* < 0.001), and longer hospital LOS 35 vs. 17 days (*p* < 0.001). 40% of VAPs were caused by MDROs. The most common organism was *Staphylococcus aureus* (28%), with almost half (48%) being methicillin resistant *Staphylococcus aureus* (MRSA).

**Conclusion:**

VAP was a common complication of patients intubated for COVID-19 pneumonia. Most patients received empiric antibiotics upon the hospital and/or ICU admission. There was a 40% incidence of multidrug resistant pneumonia. Patients who developed VAP had almost twice as long hospital and ICU LOS.

## 1. Introduction

Coronavirus Disease 2019 (COVID-19), caused by Severe Acute Respiratory Syndrome Coronavirus 2 (SARS-COV2), has claimed the lives of more than 5.4 million people around the world as of January 2022 with a mortality rate of 70.39 deaths per 100,000 population [1]. COVID-19 is primarily a pulmonary infection and up to forty-one percent of infected, hospitalized, patients require supplemental oxygen [2]. Up to fourteen percent of COVID-19 pneumonia patients develop severe respiratory disease, twelve and twenty percent require Intensive Care Unit (ICU) admission and mechanical ventilation, respectively, and sixty to seventy percent of these ICU patients develop acute respiratory distress syndrome (ARDS) [3, 4].

With a significant number of patients developing ARDS and many of them requiring ventilator support, the complication of superinfection with ventilator-associated pneumonia (VAP) is a significant concern. VAP, the most frequent intensive care unit (ICU)-acquired infection, occurs in up to 52% of patients intubated for more than 48 hours and is associated with morbidity, prolonged hospitalization, increased healthcare costs, and mortality [5].

Viral respiratory infections can be significantly complicated by the development of a secondary bacterial pneumonia. Bacterial superinfections are a major contributor to morbidity and mortality in patients with influenza pneumonia [6]. During the 2003 Hong Kong SARS outbreak, a significant incidence of VAP in SARS patients was observed with an incidence of 36.5 episodes per 1000 ventilator days [7].

There is a paucity of data informing the use of antibiotics in the management of hospitalized COVID-19 patients. The Dutch Working Party on Antibiotic Policy noted bacterial coinfection was present on admission in 3.5% of COVID-19 patients with 15% developing a secondary bacterial infection during hospitalization [8]. However, there is no, or very low-quality, evidence to guide antimicrobial management [9]. This has led to the variable practice in the use of empiric antibiotics for COVID-19 patients presenting with severe respiratory disease and creates challenges in appropriately screening patients for the development of ventilator associated pneumonia.

In this study, we report the incidence of VAP in patients intubated secondary to SARS-COV2 pneumonia, report the epidemiology and causal microorganisms for VAP, explore the association of prior empiric antibiotic administration with the identification of Multidrug Resistant Organism (MDRO) infections and evaluate the impact of VAP on patient outcomes.

## 2. Methods

### 2.1. Patient Identification and Data Collection

We conducted a multicenter, retrospective, study of all patients requiring invasive mechanical ventilation (IMV) for SARS-COV2 between March 1 and June 30, 2020, at 2 tertiary urban academic centers. This period was selected as it represented a peak wave of the pandemic in the region. Patients were identified using a registry established for quality improvement and research purposes captured in a REDCAP database. Secondary chart reviews were performed to confirm the clinical diagnosis of acute respiratory failure secondary to COVID-19. Inclusion criteria for the study were patients with polymerase chain reaction (PCR) confirmed SARS-COV2 test and a clinical diagnosis of pneumonia who received IMV. We excluded patients with a duration of IMV less than 48 hours as well as patients in whom bronchial cultures were considered to represent colonization by the treatment team (e.g., cultures growing yeast). We gathered patient demographics, comorbidities, antibiotic administration, microbiologic data (including susceptibility testing), hospital and ICU length of stay (LOS), and mortality data into a secondary Research Electronic Data Capture (REDCap) database.

### 2.2. Variables and definitions

All culture specimens were protected bronchial brush samples. Cultures were performed using the semiquantitative plating method as recommended by the American Society of Microbiology [10].

VAP was defined using the 2017 National Healthcare Safety Network classification as meeting criteria for Infection-related Ventilator Associated Complication and a positive culture with ≥1,000 colony forming units (CFU) on a protected specimen brush, occurring more than 48 hours after the initiation of mechanical ventilation [10]. Multidrug Resistant Organisms (MDRO) were identified using criteria from the European Center for Disease Prevention and Control (ECDC) and the Centers for Disease Control and Prevention (CDC), which define MDRO as nonsusceptibility to at least one agent in three or more antimicrobial categories [11].

Use of empiric antibiotics were classified into three categories: (1) preintubation empiric antibiotics given without a microbiological diagnosis; (2) postintubation empiric antibiotics given within 48 hours of initiation of IMV without a microbiological diagnosis; and (3) antibiotics given to treat microbiologically confirmed VAP based on the microbiological diagnosis. Admission order-set at both hospitals recommended the administration of ceftriaxone and azithromycin for patients admitted with a primary diagnosis of pneumonia secondary to SARS-COV2 in the first 24 hours. Subsequent de-escalation of antibiotic therapy was left up to the treatment team.

The severity of illness was assessed using both the highest SOFA score within the first 48 hours of admission and the highest daily SOFA score prior to ICU admission. SOFA score calculations were performed per the methodology described by Lambden et al. for use in randomized controlled trials [11].

### 2.3. Statistical Methods

All data were analyzed via Stata 12.1 (Stata Corp., College Station, TX, USA). Epidemiologic data comparing subjects with and without VAP as well as differences in mortality, the severity of illness, and both hospital and ICU LOS were assessed for differences using Chi^2^ and Welch's *t* tests (given unequal sample sizes and variance) as appropriate. A *p* value of 0.05 was used as the cut-off for assessing statistical significance.

Both univariate (SLR) and multivariate regression (MLR) analyses were performed for associations with mortality, ICU LOS, hospital LOS, and the development of VAP. Variables demonstrating univariate regression significance less than 0.2 were included in MLR models.

## 3. Results

One hundred-forty-eight patients with confirmed COVID-19 pneumonia requiring mechanical ventilation were identified. Seventeen patients were excluded because the duration of IMV was less than 48 hours. One hundred thirty-two patients were included in the final analyses.

The average subject age was 60, with 58% being male and seventy percent being African American. Two-thirds of patients had diabetes, hypertension, or heart disease. The average Body Mass Index (BMI) was 32.9 with 54% of patients being obese. Forty-one patients (27%) developed VAP. We found no associations between age, race, ethnicity, or comorbidities (other than a history of malignancy) with the development of VAP. Patients with VAP had a significantly higher SOFA score prior to ICU admission (14 vs. 13, *p*=0.03). Sixty percent of patients received empiric antibiotics prior to initiation of invasive mechanical ventilation (IMV), mostly on hospital admission, and 81 percent of patients received empiric antibiotics at the time of intubation. However, in both scenarios, administration of empiric antibiotics was not associated with a higher prevalence of VAP (Table 1).

### 3.1. VAP Impact on Outcomes

The prevalence of VAP in our study was 22 per 1000 days on ventilation. No difference in mortality was seen between VAP and non-VAP groups at 49% and 57%, respectively (*p*=0.4). VAP did have a significant impact on both hospital and ICU LOS. ICU LOS was 30 vs. 16 days (*p* < 0.001) for patients that developed VAP vs. non-VAP, respectively. Hospital LOS was observed at 35 vs. 17 days (*p* < 0.001) between VAP and non-VAP patients, respectively.

In univariate analyses (SLR), age, cancer, and highest SOFA score prior to ICU admission were significantly associated with mortality. However, in the final multiple logistic regression (MLR) model, age (OR 1.04, *p*=0.009), chronic liver disease (OR 3.6, *p*=−0.03), BMI (OR 0.9, *p*=0.02), and SOFA on ICU admission (OR 1.3, *p* < 0.001) were significantly associated with death. Significant associations were noted in SLR modeling between Hospital LOS and VAP (11.6 d) and age (−0.2 d) but in the subsequent MLR (linear) model, significance was only maintained by VAP (14.6 d, 95% CI = 3.9, 25.3). Similarly, in SLR modeling for ICU LOS, VAP, age, and cancer demonstrated significance, but in the subsequent MLR model, only VAP (13.4 d, 95% CI = 4.6, 22.2) and age (−0.2 d, 95% CI = −0.4, −0.02) remained statistically significant (Table 2).

In analyses for the development of VAP, only the highest SOFA score prior to ICU admission was significantly associated in SLR modeling and no one variable was significantly associated in MLR Modeling.

### 3.2. VAP Organisms

Twenty-one organisms were identified as causative factors for VAP. Two thirds of the organisms (67%) were Gram negatives and one third (33%) were Gram positives, and fifteen were polymicrobial infections. The most common organism was *Staphylococcus aureus* (28%), with almost half (48%) being methicillin resistant (MRSA). The next highest organisms were *Pseudomonas* species (19%) and *Klebsiella* species (14%). Overall, twenty VAPs (40%) were caused by MDRO. There were four incidences of VAP caused by Gram-negative enteric bacteria with an extended spectrum beta lactamase (ESBL). (Table 3).

## 4. Discussion

In this study, we found approximately one third of patients on mechanical ventilation secondary to SARS-COV2 pneumonia experience VAP. We also found that VAP is associated with a significant increase in hospital LOS and ICU LOS. The prevalence of VAP in our study far exceeds the reported 9.7% incidence of VAP in the general population [12].

Patients in the study were cared for in ICUs within two tertiary hospitals, in the same hospital network. Both hospitals implement ventilator associated pneumonia prevention protocol, as part of their mechanical ventilation order set. The protocol includes the head of bed elevation above 30 degrees, oral care every 4 hours, and chlorohexidine oral swab every 8 hours. Respiratory therapists evaluate ventilator circuits for increased secretions or clogging routinely. Both hospitals have closed ICUs with 24 hour intensivist coverage and ventilator weaning trials are done daily in patients that meet a preset criteria. These protocols were maintained during the COVID-19 pandemic. Thus, we believe there were other factors that contributed to the increased incidence of VAP.

VAP secondary to MDRO is associated with higher 28-day and overall mortality in non-SARS-COV2 patients [13]. Ninety-seven percent of the patients received one or two doses of empiric antibiotics on admission to the hospital and/or admission to the ICU. Given the high prevalence of MDRO VAP in our study, the use of empiric antibiotics without a confirmed bacterial pneumonia needs to be re-examined. At both study hospitals, protocols exist to prescribe coverage for community acquired pneumonia upon presentation for SARS-COV2 followed by early de-escalation. However, it is feasible that antimicrobial pressure of empiric antibiotics at hospital or ICU presentation contributes to a higher prevalence of VAP and development of MDRO, especially if early de-escalation is not taking place.

While we did not find that VAP independently contributes to mortality, given the available sample size and a significantly more severe state of illness in VAP patients, predicting an excess of 25% mortality [14], we believe our study was simply underpowered to identify the association of VAP and mortality among SARS-COV2. The associations between VAP and hospital and ICU LOS in COVID-19 is incredibly impactful for the healthcare system. Given the scarcity of ICU resources during the COVID-19 pandemic, a reduction in hospital and ICU LOS by 14.6 and 13.4 days, respectively, would have major implications to resource availability and cost. Patients staying longer may reflect a survivor bias which is further supported by the older and cancer patients being more likely to die, but less likely to develop VAP. VAP prevention strategies should be a major goal for optimization given both the risk for an overwhelmed healthcare system and the increased risk for overall mortality in SARS-COV2 [15]. Mitigation of hospital and ICU LOS would also likely have significant public health implications on the healthcare work force given increased concerns of burnout and the ability to maintain and equitably distributing healthcare resources [16].

We are aware of the difficulty of diagnosing a superinfection with VAP in patients with SARS-COV2 ARDS. It is challenging to clinically tease out pneumonia due to COVID-19 alone vs. concomitant bacterial superinfection as each of these clinical criteria have overlap with SARS-COV2 [17]. Relying on culture data to rule-in bacterial pneumonia may be overly specific and miss culture negative bacterial pneumonia. The increase in LOS caused by VAP suggests that proven strategies to prevent VAP should be implemented with zero tolerance in response to the demands of the COVID-19 pandemic [18]. In addition, we believe that a targeted use of antibiotics led by serial surveillance cultures and clinical markers of VAP should be considered to aid in making early diagnosis and treatment of VAP while conserving antibiotics in patients who do not have VAP.

Our study has several limitations including sample size and hospital setting (urban tertiary academic care centers). A larger study may be better positioned to parse out potential risk factors for the development of VAP in SARS-COV2 to better direct clinical practice. Likewise, a cohort study comparing the incidence of VAP in COVID-19 and non-COVID-19 ICU patients would be able to quantify the increased risk of the development of ventilator associated pneumonia.

## 5. Conclusion

Bacterial ventilator associated pneumonia is a common complication of SARS-COV2 pneumonia that arises with a much greater frequency than reported in the general intubated population. Antibiotic selection should be based on local epidemiology and knowledge of prior antibiotic exposure, favoring initial broad-spectrum antibiotics as forty percent of patients who developed VAP in this population had an infection with an MDRO. Protocols where a majority of patients receive empiric antibiotics upon hospital and ICU admission should be re-evaluated. VAP conveys major consequences on the healthcare system through a significantly increased hospital and ICU LOS risking a significant impact on the availability of critical care resources during a pandemic.

## Figures and Tables

**Table 1:** Demographic and comorbidity distributions.

	Total	VAP	No VAP	*p* ^*∗*^
N	148	41	107	
Age, mean (years)	60	58.6	60.5	0.5
Sex, %				
Male, *n* (%)	86 (58)	26 (63)	60 (56)	0.4
Female, *n* (%)	62 (42)	15 (37)	47 (44)	0.4
Race, %				
Black	99 (70)	24 (62)	75 (74)	0.2
White	10 (7)	4 (10)	6 (6)	0.4
Native American	1 (1)	0 (0)	1 (1)	—
Other	31 (21)	11 (28)	20 (20)	0.3
Ethnicity, %				
Hispanic	30 (20)	12 (32)	18 (19)	0.09
Nonhispanic	102 (69)	26 (68)	76 (81)	0.4
Comorbidities, %				
Cancer	29 (20)	4 (10)	25 (23)	0.03
Diabetes mellitus	86 (58)	27 (66)	59 (55)	0.2
Heart disease	95 (64)	29 (71)	66 (62)	0.3
Hypertension	99 (67)	25 (61)	74 (69)	0.5
Immune disorder	18 (12)	4 (10)	14 (13)	0.6
Chronic kidney disease	23 (16)	9 (22)	14 (13)	0.2
Chronic liver disease	24 (16)	9 (22)	15 (14)	0.2
Prior CVA	7 (5)	3 (7)	4 (3)	0.7
BMI, mean	32.9	34.2	32.4	0.3
Highest SOFA score within 48 hours of admission (mean)	6	6	5	0.4
Highest SOFA score prior to ICU admission (mean)	13	14	13	0.03
Empiric antibiotic prior to IMV (%)	60 (41)	24 (59)	60 (56)	0.8
Empiric antibiotic within 48 hours of IMV (%)	120 (81)	36 (88)	84 (79)	0.2
Discharge disposition, *n* (%)				
Mortality	81 (55)	20, (49)	61, (57)	0.4^*∗∗*^
Home	40 (27)	6, (15)	34, (32)	0.04
Skilled facility	28 (19)	15, (37)	13, (12)	<0.001
ICU LOS, mean (d)	20	30	16	<0.001^*∗∗∗*^
Hospital LOS, mean (d)	22	35	17	<0.001^*∗∗∗*^

^*∗*^ *p* values by chi-squared or welch's *t*-test. ^*∗∗*^*p* value calculated by chi-squared; ^*∗∗∗*^*p* value calculated by welch's *t*-test *P* values by *t*-test for VAP by each variable. VAP–ventilator associated pneumonia; CVA–cerebrovascular accident; BMI–body mass index; SOFA–sequential organ failure assessment; IMV–invasive mechanical ventilation; ICU–intensive care unit; LOS–length of stay.

**Table 2:** Multivariate (MLR) regression analyses for factors associated with VAP.

	Mortality^*∗*^		Hospital LOS^*∗∗*^ (days)		ICU LOS^*∗∗∗*^ (days)	
	MLR OR	*p*	MLR coef	*p*	MLR coef	*p*
VAP			14.6	0.008	13.4	0.003
Age	1.05	0.01	−0.2	0.08	−0.2	0.03
PMH						
Cancer	2.7	0.09	−0.9	0.8	−1.4	0.6
Heart disease	1.3	0.5				
BMI	0.9	0.02	0.02	0.9	0.04	0.7
Chronic liver disease	3.6	0.03				
DM			4.8	0.7	4.1	0.7
Immunosuppression					−6.7	0.048
African American			−1.4	0.6	−0.9	0.7
Antibiotics (intubation)			4.7	0.1	4.0	0.1
Antibiotics (at final diagnosis)			1.4	0.8	−2.1	0.6
SOFA (hospital admit)			−0.2	0.4	−0.2	0.3
SOFA (ICU admit)	1.3	<0.001				

Only variables with univariate regression *p* < 0.2 were included in multi-logistic regression. VAP–ventilator associated pneumonia; LOS–length of stay; MLR–multivariate regression; PMH–past medical history; BMI–body mass index; Dm–diabetes mellitus; SOFA–sequential organ failure assessment; ICU–intensive care unit.

**Table 3:** Frequency of VAP organisms and MDROs.

VAP species	Count	MDRO
*Staphylococcus aureus*	14 (28%)	6 (12%)
*Klebsiella* spp	10 (20%)	2 (4%)
*Pseudomonas* spp	8 (16%)	8 (16%)
*Escherichia coli*	4 (8%)	1 (2%)
*Stentrophomonas maltophilia*	2 (4%)	2 (4%)
*Serratia* spp	2 (4%)	1 (2%)
*Haemophilus influenzae*	2 (4%)	
*Moraxella catarrhalis*	2 (4%)	
*Citrobacter* spp	2 (4%)	
*Enterobacter* spp	1 (2%)	
*Streptococcus pneumoniae*	2 (4%)	
*Streptococcus agalactiae*	1 (2%)	
*Total*	50	20 (40%)

VAP–ventilator associated pneumonia; MDRO–multi-drug resistant organism; Spp–species.

## Data Availability

The dataset used to support the findings of this study are available from the corresponding author upon request.

## References

[1] (2020). WHO COVID-19 dashboard. https://covid19.who.int/table%20on%201/12/2022.

[2] Guan W. J., Ni Z. Y., Hu Y. (2020). Clinical characteristics of coronavirus disease 2019 in China. *New England Journal of Medicine*.

[3] Phua J., Weng L., Ling L. (2020). Intensive care management of coronavirus disease 2019 (COVID-19): challenges and recommendations. *The Lancet Respiratory Medicine*.

[4] Richardson S., Hirsch J. S., Narasimhan M. (2020). Presenting characteristics, comorbidities, and outcomes among 5700 patients hospitalized with COVID-19 in the New York city area. *JAMA*.

[5] Charles M. P., Kali A., Easow J. M. (2014). Ventilator-associated pneumonia. *Australasian Medical Journal*.

[6] Prasso J. E., Deng J. C. (2017). Postviral complications: bacterial pneumonia. *Clinics in Chest Medicine*.

[7] Yap F. H. Y., Gomersall C. D., Fung K. S. C. (2004). Increase in methicillin-resistant *Staphylococcus aureus* acquisition rate and change in pathogen pattern associated with an outbreak of severe acute respiratory syndrome. *Clinical Infectious Diseases*.

[8] Karami Z., Knoop B. T., Dofferhoff A. S. M. (2021). Few bacterial co-infections but frequent empiric antibiotic use in the early phase of hospitalized patients with COVID-19: results from a multicentre retrospective cohort study in The Netherlands. *Infectious Diseases*.

[9] Sieswerda E., de Boer M. G. J., Bonten M. M. J. (2021). Recommendations for antibacterial therapy in adults with COVID-19-an evidence based guideline. *Clinical Microbiology and Infections*.

[10] Baselski V. S., el-Torky M., Coalson J. J., Griffin J. P. (1992). The standardization of criteria for processing and interpreting laboratory specimens in patients with suspected ventilator-associated pneumonia. *Chest*.

[11] Magiorakos A. P., Srinivasan A., Carey R. B. (2012). Multidrug-resistant, extensively drug-resistant and pandrug-resistant bacteria: an international expert proposal for interim standard definitions for acquired resistance. *Clinical Microbiology and Infections*.

[12] Lambden S., Laterre P. F., Levy M. M., Francois B. (2019). The SOFA score-development, utility and challenges of accurate assessment in clinical trials. *Critical Care*.

[13] Metersky M. L., Wang Y., Klompas M., Eckenrode S., Bakullari A., Eldridge N. (2016). Trend in ventilator-associated pneumonia rates between 2005 and 2013. *JAMA*.

[14] Parker C. M., Kutsogiannis J., Muscedere J. (2008). Ventilator-associated pneumonia caused by multidrug-resistant organisms or *Pseudomonas aeruginosa*: prevalence, incidence, risk factors, and outcomes. *Journal of Critical Care*.

[15] Ferreira F. L., Bota D. P., Bross A., Mélot C., Vincent J. L. (2001). Serial evaluation of the SOFA score to predict outcome in critically ill patients. *JAMA*.

[16] Pasquariello P., Stranges S. (2020). Excess mortality from COVID-19: a commentary on the Italian experience. *International Journal of Public Health*.

[17] Miller I. F., Becker A. D., Grenfell B. T., Metcalf C. J. E. (2020). Disease and healthcare burden of COVID-19 in the USA. *Nature Medicine*.

[18] François B., Laterre P. F., Luyt C. E., Chastre J. (2020). The challenge of ventilator-associated pneumonia diagnosis in COVID-19 patients. *Critical Care*.

[19] Yokoe D. S., Anderson D. J., Berenholtz S. M. (2014). A compendium of strategies to prevent healthcare-associated infections in acute care hospitals: 2014 updates. *American Journal of Infection Control*.

